# An Accelerated Maximum Flow Algorithm with Prediction Enhancement in Dynamic LEO Networks

**DOI:** 10.3390/s25082555

**Published:** 2025-04-17

**Authors:** Jiayin Sheng, Xinjie Guan, Fuliang Yang, Xili Wan

**Affiliations:** College of Computer and Information Engineering, Nanjing Tech University, Nanjing 211816, China; jiayinsheng@njtech.edu.cn (J.S.); yfl@njtech.edu.cn (F.Y.); xiliwan@njtech.edu.cn (X.W.)

**Keywords:** satellite networks, time-expanded graph, max flow routing, algorithms with predictions

## Abstract

Efficient data transmission in low Earth orbit (LEO) satellite networks is critical for supporting real-time global communication, Earth observation, and numerous data-intensive space missions. A fundamental challenge in these networks involves solving the maximum flow problem, which determines the optimal data throughput across highly dynamic topologies with limited onboard energy and data processing capability. Traditional algorithms often fall short in these environments due to their high computational costs and inability to adapt to frequent topological changes or fluctuating link capacities. This paper introduces an accelerated maximum flow algorithm specifically designed for dynamic LEO networks, leveraging a prediction-enhanced approach to improve both speed and adaptability. The proposed algorithm integrates a novel energy-time expanded graph (e-TEG) framework, which jointly models satellite-specific constraints including time-varying inter-satellite visibility, limited onboard processing capacities, and dynamic link capacities. In addition, a learning-augmented warm-start strategy is introduced to enhance the Ford–Fulkerson algorithm. It generates near-optimal initial flows based on historical network states, which reduces the number of augmentation steps required and accelerates computation under dynamic conditions. Theoretical analyses confirm the correctness and time efficiency of the proposed approach. Evaluation results validate that the prediction-enhanced approach achieves up to a 32.2% reduction in computation time compared to conventional methods, particularly under varying storage capacity and network topologies. These results demonstrate the algorithm’s potential to support high-throughput, efficient data transmission in future satellite communication systems.

## 1. Introduction

In recent years, Low Earth Orbit (LEO) satellite networks have transformed global communications, enabling real-time, high-bandwidth applications across fields such as Earth observation, disaster management, remote sensing, and global internet access. These networks, exemplified by large-scale projects like the SpaceX Starlink [1], Stallion’s OneWeb [2], and Amazon’s Kuiper [3], now comprise thousands of satellites working to deliver extensive, low-latency coverage worldwide [4]. As demand for high-throughput, real-time data services increases, efficient data transmission across these dynamic and resource-constrained networks becomes a central challenge. The integration of LEO networks with 5G, 6G, and IoT infrastructures further amplifies this need while introducing new architectural complexity [5].

A fundamental challenge in LEO networks is efficiently handling these large-scale, dynamic data flows. Satellites move rapidly, resulting in frequent changes in connectivity between nodes and ground stations. The connectivity properties of large-scale LEO constellations have been recently analyzed using percolation theory [6], providing insights into how network topology influences communication reliability and data flow. Additionally, limited onboard data processing capability and fluctuating link capacities impose further constraints on optimizing data flow. At the core of this challenge is the maximum flow problem, a classic network optimization problem that seeks to maximize data throughput from source nodes to destination nodes across a network. Solving this problem efficiently is essential for sustaining real-time data delivery in large-scale satellite systems.

**Figure 1:**
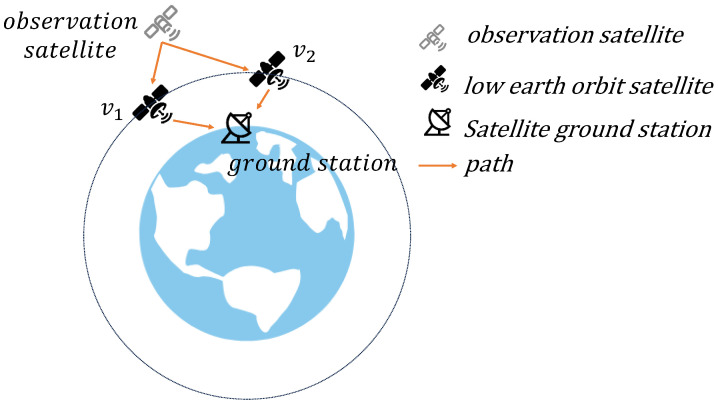
A demo example of an observation satellite transmitting data to an Earth ground station via relay satellites in a LEO constellation.

To illustrate, consider a representative scenario that involves an observation satellite transmitting data to a distant ground station. Due to movement in the constellation, the transmission path often includes multiple intermediate relay satellites. Figure 1 shows a simplified example of data flow; the observation satellite (node *s*) transmits data to the ground station (node *g*) through relay satellites (nodes $v_{1}$ and $v_{2}$) along two different paths, $s-v_{1}-g$ and $s-v_{2}-g$. Each link has limited capacity, and as satellites move, connectivity changes, requiring frequent recomputation of the optimal data path. In this scenario, the maximum flow problem involves calculating the highest data throughput from *s* to *g* across all available paths.

In static networks, maximum flow calculations can often be handled with traditional algorithms like Ford–Fulkerson [7] or Dinic’s [8]. However, in LEO networks, every topological change, whether due to orbital motion, link failure, or energy constraints, leads to a full recomputation, limiting responsiveness in real-time applications.

To address these limitations, recent research has turned to predictive and adaptive techniques for optimizing maximum flow calculations. By leveraging historical data and predictive modeling, these approaches aim to warm-start flow calculations [9], reducing the need for costly recalculations as the network evolves. Reinforcement learning has also been explored to optimize data transmission in LEO networks, particularly for congestion control and efficient routing [10]. Inspired by these approaches, this paper introduces a novel accelerated maximum flow algorithm specifically tailored to the unique challenges of dynamic LEO networks with high data throughput requirements.

The proposed approach combines a predictive warm-start mechanism with an extended application of the energy-time expanded graph (e-TEG) model [11], offering a solution that operates efficiently under the dual constraints of dynamic topologies and limited processing capability of satellites. Unlike traditional maximum flow algorithms that require recalculations with each network change, our method begins with a predictive warm-start informed by past and anticipated network states, minimizing computational demands and allowing rapid response to network shifts without recalculating from scratch.

In addition to predictive warm-starting, we incorporate a learning-augmented component that adapts flow estimations based on historical network patterns. Machine learning techniques, including graph embedding-based reinforcement learning, have recently been explored to optimize routing and path selection in large-scale LEO networks [12]. Inspired by these advancements, our approach leverages historical network behavior to enhance flow predictions, further reducing the need for recalculations and improving computational efficiency. The combination of predictive initialization, e-TEG modeling, and learning-augmented adaptability forms a high-performance framework for data-intensive applications in LEO networks.

The main contributions of this paper are summarized as follows:

(1) We introduce a novel predictive warm-start approach for maximum flow calculations in dynamic LEO networks. The method uses prior and anticipated network states to initialize the algorithm closer to optimal solutions, significantly reducing computation time.
(2) The proposed algorithm incorporates a learning-augmented component that uses historical network patterns to inform flow predictions. This feature allows the algorithm to dynamically adjust to network shifts with minimal recalculations, enhancing its robustness and efficiency in environments with frequent connectivity changes.
(3) We provide a theoretical analysis of the predictive flow algorithm’s feasibility and computational efficiency, supported by extensive empirical validation. Experimental results confirm the algorithm’s robustness and effectiveness across varied network and hardware conditions in LEO environments.

The remainder of this paper is organized as follows. Section 2 reviews the related work on maximum flow computation in LEO satellite networks and the application of predictive techniques to enhance the Ford–Fulkerson algorithm. Section 3 describes the system model and formulates the maximum flow problem in LEO networks. Section 4 details the proposed prediction algorithm designed for LEO networks and the warm-start Ford–Fulkerson accelerated algorithm. Section 5 validates the performance of both algorithms through extensive evaluations. Finally, Section 6 concludes this work and discusses potential research directions.

## 2. Related Work

Advancements in satellite network modeling and maximum flow algorithms have laid the groundwork for addressing the challenges of dynamic and capacity-constrained LEO satellite networks. This section reviews the main development and application of TEG and e-TEG models in satellite networks, important advancements in maximum flow algorithms, including predictive and learning-augmented approaches, and the integration of the software-defined networking (SDN) framework into the satellite constellation.

### 2.1. TEG and e-TEG in LEO Satellite Networks

TEG has emerged as a powerful tool for modeling the dynamic topology of satellite networks. By representing the network’s state at different time points as a series of snapshots, TEG provides an effective means for analyzing and optimizing flow transmission in satellite networks [13]. The dynamic nature of satellite networks, characterized by time-varying topologies and dynamic bandwidth, poses challenges for traditional network analysis techniques. TEG addresses these challenges by capturing the network’s evolution over time, allowing researchers to formulate and solve problems related to network capacity, routing strategies, and flow optimization. The application of TEG in satellite networks extends beyond flow optimization. It has been used for network capacity analysis, enabling researchers to determine the upper bound of transmission capacity in a given satellite network.

While TEG provides a robust framework for analyzing satellite networks, it does not account for the limited energy resources of satellites, which are critical in satellite operations. To address this limitation, researchers have extended TEG to e-TEG [11], which incorporates energy constraints into the model, providing a more accurate representation of satellite networks with energy-limited satellites. In e-TEG, virtual arcs are introduced to model energy consumption, with capacity bounds representing available energy resources.

The e-TEG model has enabled researchers to address the energy-limited maximum flow problem, where the objective is to maximize data throughput while ensuring that energy consumption remains within the satellite’s limited resources. Algorithms leveraging temporal augmenting paths [14] have shown significant improvements in throughput and energy efficiency, making e-TEG a foundational tool for optimizing data transmission in LEO satellite networks. In addition to energy constraints, dynamic resource allocation strategies have been developed to address the high mobility and dynamic characteristics of LEO satellites, improving resource utilization and system throughput [15].

### 2.2. Advancements in Maximum Flow Algorithms

The Ford–Fulkerson method [7] is a classical approach to computing maximum flow in networks. Variants of the maximum flow algorithm have introduced path-finding optimizations to improve runtime. For example, the Edmonds–Karp algorithm [16] uses breadth-first search (BFS) to identify the shortest augmenting path, while Dinic’s algorithm [8] employs a layered network structure to accelerate convergence in certain types of networks. These methods have been widely used in various applications, achieving practical efficiency in static networks.

Recently, learning-augmented techniques have emerged as a promising direction for improving runtime by leveraging prior knowledge. For instance, Polak et al. [9] proposed a learning-augmented maximum flow algorithm that uses predicted flow values to accelerate computation. Their method achieves a runtime of O(mη), where *m* is the number of edges and η is the $l_{1}$ prediction error. However, these predictions do not guarantee adherence to edge capacity constraints, limiting their direct applicability to certain network problems.

While other works, such as [17], addressed specific cases like 0–1 min-cost flow, their techniques rely on heavily constrained problem setups (e.g., binary capacities) and do not generalize to the broader maximum flow problem. Similarly, frameworks for warm-starting algorithms [18] are limited to specific problem types, such as L-convex optimization, and are not directly applicable to flow networks. Ref. [19] demonstrated the use of predictions to enhance the runtime of generalized sorting.

Additionally, research on leveraging data-driven approaches for algorithm optimization has gained traction. For instance, a study presented in [20] explored the integration of machine learning predictions within traditional algorithmic frameworks, with a focus on improving efficiency. Furthermore, ref. [21] discussed how predictive models can be utilized to inform and accelerate algorithmic processes, particularly in the domain of network flow problems. In a related area, data-driven algorithm design, e.g., [22,23], has emerged as a promising direction for leveraging data to optimize algorithm performance, further highlighting the potential of incorporating machine learning into traditional algorithmic paradigms.

Beyond these advancements, recent research efforts have focused on enhancing the efficiency and versatility of maximum flow algorithms within dynamic and constrained environments. Notably, Sherman [24] and Kelner et al. [25] developed fast algorithms for computing approximate maximum flows, offering substantial speed improvements for large-scale networks. Furthermore, Chen et al. [26] introduced an algorithm that computes the maximum flow in nearly linear time. However, it is not yet feasible for practical applications in the real world due to its restrictive data assumptions, complex implementation, and inability to fully meet the demands of high real-time and highly dynamic scenarios. Altner and Ergun [27] empirically demonstrated the potential to accelerate the push-relabel algorithm by employing warm-starts on similar networks, underscoring the opportunity to leverage prior solutions for faster computations.

While TEG and e-TEG have laid a solid foundation for modeling dynamic satellite networks, existing algorithms for solving maximum flow problems in these models do not fully leverage the potential of predictive acceleration. Current warm-starting techniques, though promising, either focus on restricted problem setups or lack integration with dynamic, capacity-constrained environments like e-TEG.

To provide a clearer understanding of the distinctions and connections among these models and algorithms, Table 1 provides a systematic comparison of the TEG, e-TEG models, and representative maximum flow algorithms in terms of their characteristics, advantages, and limitations.

### 2.3. Software-Defined Satellite Networks

To enhance the adaptability of satellite networks and reduce the computational load on individual satellites, the SDN framework has been seamlessly integrated into the satellite constellation, giving rise to the software-defined satellite network (SDSN) [28,29,30]. By capitalizing on the centralized management and programmability features of SDN, the SDSN establishes a foundation for holistic satellite network management. This is achieved through the separation of the control plane from the LEO forwarding plane, which enables more sophisticated monitoring of network conditions and more efficient resource allocation, as evidenced in [30].

Bao et al. [31] presented a comprehensive review of an SDN-centric satellite network architecture. This architecture uses at least three Geostationary Earth Orbit (GEO) satellites as controllers to improve both operational efficiency and fine-grained control across the entire satellite constellation. To overcome the limitations of existing satellite communication systems, such as slow configuration and poor quality of service (QoS) guarantees, the software-defined framework for integrated space-terrestrial satellite communication has introduced flexible traffic engineering strategies and optimized QoS mechanisms.

These advancements are further explored in related studies. Ref. [32] conducted a detailed analysis of SDN-based satellite networks with the aim of improving performance, while [33] delved into leveraging SDN for dynamic resource management within satellite constellations. Moreover, the integration of SDN with satellite networks, which significantly improves flexibility and scalability, is extensively discussed in [34], highlighting the potential of SDSNs in space communications.

## 3. System Model and Problem Formulation

In this section, we present the system model and formally define the maximum flow problem within LEO satellite networks. We first introduce the general formulation of the maximum flow problem, which serves as the foundation for modeling data throughput in satellite networks. Then, considering the dynamic and resource-constrained nature of these networks, several practical limitations are accounted including visibility conditions that govern communication links, processing capacity limits of satellites, and bandwidth availability for data transmission. Additionally, we explore how the dynamic evolution of the network topology and potential network failures impact flow optimization. Together, these factors shape the problem of maximizing flow in real-world LEO networks, forming the basis for our proposed solution.

### 3.1. Definitions and Problem Formulation

Considering a LEO satellite network comprises a source satellite $s_{1}$, a set of relay satellites $\left\{r_{1},r_{2},\ldots ,r_{N}\right\}$, and a ground station $g_{1}$, the primary objective is to maximize the flow of spatial data from the source to the ground station within a specified time horizon $T=[t_{0},t_{k})$. Data transmission can occur directly or via relay satellites, with operations constrained by the storage capacity of each satellite, the time-varying transmission capacity of communication links, and the energy budget available to each satellite.

The time horizon *T* is divided into *k* discrete time slots, denoted as $\left\{\tau_{1},\tau_{2},\ldots ,\tau_{k}\right\}$, where each time slot $\tau_{h}=\left[t_{h-1},t_{h}\right)$ represents a static snapshot of the network. Satellites in the network are equipped with limited storage capacity, which restricts the amount of data that can be buffered at any given time. Formally, the total data stored at a satellite $v_{i}$ during the time horizon must not exceed its buffer capacity $S_{v_{i}}$. It ensures that the satellite can manage incoming and outgoing data efficiently and reflects the operational limitations inherent in satellite hardware.

Communication between satellites and the ground station relies on directed communication links with time-dependent transmission capacities. For a link between two nodes, represented as $\left(v_{i},v_{j}\right)$, the volume of data transmitted during a time slot $\tau_{h}$ cannot exceed the capacity $C_{v_{i},v_{j}}^{h}$. These capacities vary across time slots due to the dynamic nature of LEO networks, which experience frequent topology changes as satellites move along their orbits.

Energy is another critical resource in LEO satellite networks. Each satellite has a finite energy budget, denoted by $E_{v_{i}}$, which governs its ability to transmit and process data throughout the time horizon. As satellites consume energy to maintain communication links, buffer data, and relay information, the total energy consumed by a satellite over all time slots must remain within its budget.

The goal of the network is to maximize the total data flow *F* from the source $s_{1}$ to the ground station $g_{1}$ while satisfying the constraints imposed by storage, transmission capacity, and satellite energy. The total flow *F* is calculated as the sum of all data transmitted from $s_{1}$ to $g_{1}$ across all time slots. Formally, the optimization problem can be expressed as follows:

(1) $$F=\sum_{h=1}^{k}\sum_{(s_{1},\mu )\in E}f_{s_{1},\mu }^{h}$$

where $f_{s_{1},\mu }^{h}$ represents the data flow from $s_{1}$ to its neighboring node μ during the time slot $\tau_{h}$. The flow must respect the constraints associated with storage, transmission capacity, and energy, ensuring the solution remains feasible under the physical and operational limitations of the network, as formulated below:

(2) $$0\leq f_{v_{i},v_{j}}^{h}\leq C_{v_{i},v_{j}}^{h},\;\forall i,j\in \left[1,N\right],\forall h\in \left[1,k\right]$$

(3) $$\sum_{h=1}^{k}\sum_{j:(v_{j},v_{i})\in E}f_{v_{j},v_{i}}^{h}\leq S_{v_{i}},\;\forall i\in \left[1,N\right]$$

(4) $$\sum_{h=1}^{k}E_{v_{i}}^{h}\leq E_{v_{i}},\;\forall i\in \left[1,N\right]$$

To align with practical satellite operations, we consider a variety of constraints that limit the network’s ability to handle data flow in Section 3.2 and Section 3.3.

### 3.2. Visibility Constraints of Satellite Networks

In LEO satellite networks, communication between any pair of nodes, that is, satellites, or ground stations, is governed by geometric visibility conditions. A data transmission link can only be established if two nodes have an unobstructed line-of-sight (LoS) connection during a given time slot [35]. Since LEO satellites move rapidly along their orbits, the availability of communication links varies over time, requiring a time-dependent representation of network connectivity.

For a direct communication link between two nodes $v_{i}$ and $v_{j}$ to be feasible, the minimum elevation angle $\theta_{min}$ defines the threshold for communication. The maximum allowable elevation angle $\delta_{ij}$ for establishing a communication link between two nodes is determined by the Earth’s curvature and the altitude of the satellites. This angle can be calculated as follows for satellite-to-ground and satellite-to-satellite links:

(5) $$\delta_{ij}=90^{\circ }-\theta_{\min}-\arcsin\left(\frac{r_{E}+h_{i}}{r_{E}+h_{j}}\cdot \sin\left(90^{\circ }+\theta_{\min}\right)\right)$$

where $r_{E}$ is the Earth’s radius, and $h_{i}$ and $h_{j}$ are the altitudes of satellites $v_{i}$ and $v_{j}$, respectively, assuming that $v_{i}$ is at a relatively higher altitude than $v_{j}$.

For satellite-to-satellite communication, the visibility condition is satisfied when the angular $∠OPv_{j}$ does not exceed the maximum allowable link angle δij. The angle $∠OPv_{j}$ is defined as the angular separation between the center of the Earth *O*, an intermediate intersection point, *P*, and the receiving satellite, $v_{j}$. The intersection point, *P*, is determined along the line connecting *O* and $v_{j}$ such that it satisfies the minimum elevation angle constraint required for satellite visibility. Using trigonometric relationships, this angle is computed as follows:

(6) $$∠OPv_{j}=2\arcsin\left(\frac{\left|Pv_{j}\right|}{2(r_{E}+h_{j})}\right)\leq \delta_{ij}$$

where $\left|Pv_{j}\right|$ is the Euclidean distance between the intersection point *P* and the satellite $v_{j}$.

For both satellite-to-satellite and satellite-to-ground links, communication is only possible if the angular separation between the two nodes is less than or equal to $\delta_{ij}$, ensuring LoS. This condition is mathematically represented as follows:

(7) $$\left(v_{i},v_{j}\right)\in E^{h}\;\mathrm{iff}\;\theta_{ij}^{h}\leq \delta_{ij}$$

where $E^{h}$ represents the set of feasible links at time slot $\tau_{h}$, and $\theta_{ij}^{h}$ denotes the angular separation between the two nodes $v_{i}$ and $v_{j}$ at time slot $\tau_{h}$.

Thus, these visibility constraints are fundamental for determining which links are available for data transmission at any given time, ensuring that only valid communication paths are used. These constraints are crucial when calculating the maximum flow in the network, as they directly limit the set of feasible paths through which data can be transmitted.

### 3.3. Capability Constraints of Satellites

For any satellite node $r_{i},i\in \left[1,N\right]$, its processing capacity, denoted as $C_{i}$, defines the maximum amount of data it can handle within a given time period. This capacity is determined by several factors, including the inherent processing capacity $f_{i}$ of the node and its available energy reserves $E_{i}$ for data processing and forwarding. The energy consumption per unit of processing *p* further constrains the amount of data the satellite can handle, which is mathematically captured by the following equation:

(8) $$C_{i}=\left(min\left(f_{i},\frac{E_{i}}{p}\right)\right)\times T^{\zeta }$$

where $T^{\zeta }$ accounts for the temporal dynamics and scaling effects associated with data processing over the time period, *T*. Therefore, the data volume processed on the satellite $r_{i}$ during the time horizon cannot exceed $C_{i}$, considering its processing capacity and available energy.

**Figure 2:**
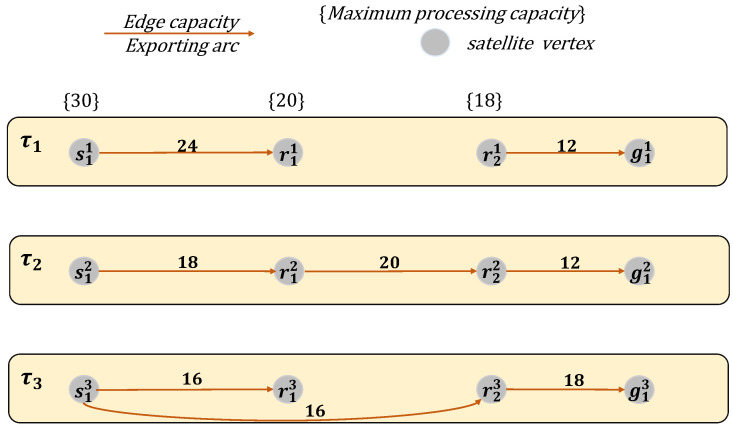
Transmission paths over time slots and satellite processing capacity constraints as represented in the modeled satellite network in Figure 1.

As discussed in Section 3.1, the processing capacity constraints, combined with visibility constraints define the available communication links and data transmission paths across the satellite network. We abstract the transmission paths across different time slots, taking into account both visibility and the satellite’s maximum processing capacity, as illustrated in Figure 1. The time-varying constraints and resulting transmission capacities are presented in Figure 2, visually capturing the network’s operational limits over time.

### 3.4. Construction of the e-TEG Satellite Network

To address data flow optimization in dynamic, resource-constrained LEO satellite networks, we construct the e-TEG framework. It captures network topology and resource constraints across discrete time slots, enabling time-aware maximum flow computation.

The e-TEG maps satellites, ground stations, and their communication links into an expanded graph that reflects temporal and resource-dependent relationships. The node set $V^{*}$ and the arc set $A^{*}$ of the e-TEG are derived as follows.

First, for each satellite and the ground station, a distinct node is created for every time slot $\tau_{h}$, where 1≤h≤k. For a satellite $v_{i}$, this results in a series of temporal replicas $\left\{v_{i}^{1},v_{i}^{2},\cdots ,v_{i}^{k}\right\}$, each corresponding to its state in a specific time slot. Similarly, the source satellite $s_{1}$ and the ground station $g_{1}$ are represented as $\left\{s_{1}^{1},s_{1}^{2},\cdots ,s_{1}^{k}\right\}$ and $\left\{g_{1}^{1},g_{1}^{2},\cdots ,g_{1}^{k}\right\}$, respectively. Virtual nodes $V_{t}$ and $V_{a}$ are introduced to model the sending and receiving states of data flow at each time slot, completing the construction of the node set:

(9) $$V^{*}=V_{s}\cup V_{r}\cup V_{g}\cup V_{t}\cup V_{a}$$

where $V_{s}$, $V_{r}$, and $V_{g}$ correspond to the source, relay, and ground station nodes across time slots, respectively.

Next, arcs are added to model the data flow and its constraints. For each node $v_{i}^{h}$, an arc $\left(v_{i}^{h},v_{i}^{h+1}\right)$ is created to represent data caching across consecutive time slots. These arcs form the caching set $A_{c}$. Additionally, virtual arcs $\left(v_{i}^{h},{\hat{v}}_{i}^{h}\right)$ and $\left({\tilde{v}}_{i}^{h},v_{i}^{h}\right)$ are added to connect physical nodes with their virtual sending and receiving counterparts. These virtual arcs, collectively denoted by $A_{s}$ and $A_{r}$, enable the modeling of data flow within a single time slot.

Finally, transmission arcs $\left({\hat{v}}_{i}^{h},{\tilde{v}}_{j}^{h}\right)$ are introduced to represent feasible data transfer between nodes $v_{i}^{h}$ and $v_{j}^{h}$ within a time slot $\tau_{h}$. These arcs, denoted as $A_{e}$, are only added if a direct communication link exists between $v_{i}$ and $v_{j}$ during $\tau_{h}$, subject to LoS constraints. The complete arc set is, therefore, expressed as follows:

(10) $$A^{*}=A_{c}\cup A_{s}\cup A_{r}\cup A_{e}$$

Each arc in $A^{*}$ is assigned a capacity to reflect the network’s constraints. For transmission arcs $\left({\hat{v}}_{i}^{h},{\tilde{v}}_{j}^{h}\right)\in A_{e}$, the capacity $C_{{\hat{v}}_{i}^{h},{\tilde{v}}_{j}^{h}}^{*}$ is calculated as follows:

(11) $$C_{{\hat{v}}_{i}^{h},{\tilde{v}}_{j}^{h}}^{*}=\int_{\tau_{h}}R_{v_{i}^{h},v_{j}^{h}}\left(t\right)\;dt$$

where $R_{v_{i}^{h},v_{j}^{h}}\left(t\right)$ is the data transmission rate between $v_{i}$ and $v_{j}$ during $\tau_{h}$. For caching arcs $\left(v_{i}^{h},v_{i}^{h+1}\right)\in A_{c}$, the capacity is set to the buffer size $S_{v_{i}}$. Virtual arcs $A_{s}$ and $A_{r}$ are assigned infinite capacity to ensure they do not impose additional constraints.

In the e-TEG model, each satellite $v_{i}^{h}$ is associated with a processing capacity $C_{i}^{h}$ at the end of each time slot $\tau_{h}$. This value represents the maximum amount of data the satellite can process during that time, taking into account its available energy and hardware limitations. As data processing progresses, this capacity is dynamically updated to reflect energy consumption, ensuring that the total data processed across all time slots does not exceed the satellite’s energy budget:

(12) $$\sum_{h=1}^{k}C_{v_{i}}^{h}\leq C_{v_{i}},\;\forall i\in \left[1,N\right]$$

**Figure 3:**
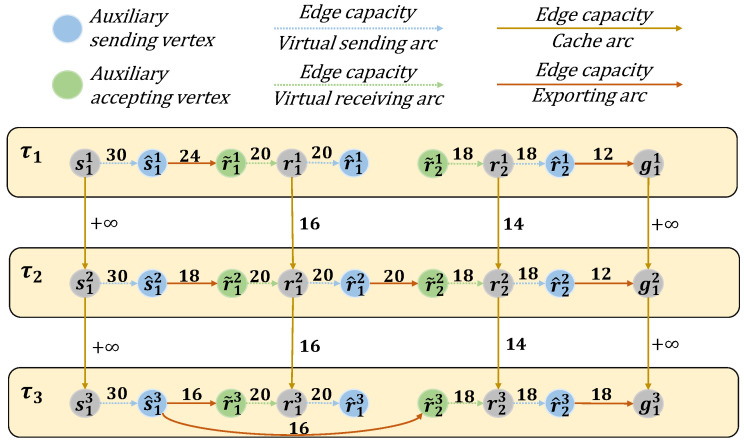
e-TEG model based on one source satellite, two LEO satellites, and one ground station.

By constructing the e-TEG with temporal replicas, virtual nodes, and resource-constrained arcs, the framework accurately captures the dynamic and resource-constrained nature of LEO satellite networks. This representation enables efficient computation of maximum flow solutions while adapting to frequent topology changes and operational limitations. Figure 3 gives a demo example of the e-TEG model for a LEO with one source, two relays, and one destination ground station in three time slots.

### 3.5. Update Mechanism of the Residual Network

To efficiently compute maximum flow in a dynamic LEO satellite network, the residual network must be continuously updated to reflect changes in network topology and resource constraints [36]. As the time horizon progresses, the network evolves, with new communication links emerging, existing links vanishing, or resource capacities fluctuating. To account for these changes, we employ a residual network update mechanism integrated with the e-TEG framework. This mechanism allows for the dynamic expansion and adjustment of the network model, ensuring that maximum flow computations remain valid and up-to-date.

Initially, the e-TEG is constructed based on the network’s state during the first time slot $\tau_{1}$. This initial representation serves as the starting residual network, encapsulating the residual capacities of nodes and edges. As the solving process progresses, the e-TEG is incrementally expanded by incorporating new snapshots of the network corresponding to subsequent time slots $\left\{\tau_{2},\tau_{3},\cdots ,\tau_{k}\right\}$. For each time slot $\tau_{i}$, the residual network is updated to reflect the current state of the network, including changes in node and edge capacities.

To achieve this, a snapshot graph $G_{i}^{\prime }=\left(V_{i}^{\prime },A_{i}^{\prime }\right)$ is constructed for each time slot $\tau_{i}$, capturing all nodes, edges, and their associated capacities during that interval. The snapshot graph is then integrated into the existing e-TEG to form the updated graph $G_{i}=\left(V_{i}^{*},A_{i}^{*}\right)$, where we have the following:

(13) $$V_{i}^{*}=V_{i-1}^{*}\cup V_{i}^{\prime },\;A_{i}^{*}=A_{i-1}^{*}\cup A_{i}^{\prime }$$

This process ensures that the e-TEG evolves to represent the network’s state from $\tau_{1}$ to $\tau_{i}$.

During the update process, the capacities of edges in the residual network are adjusted to reflect the flow already transmitted and the newly available capacities. For an edge $\left({\hat{v}}_{i}^{h},{\tilde{v}}_{j}^{h}\right)\in A^{*}$, the updated capacity $C_{{\hat{v}}_{i}^{h},{\tilde{v}}_{j}^{h}}^{\prime }$ is given by the following:

(14) $$C_{{\hat{v}}_{i}^{h},{\tilde{v}}_{j}^{h}}^{\prime }=C_{{\hat{v}}_{i}^{h},{\tilde{v}}_{j}^{h}}+F_{{\hat{v}}_{i}^{h},{\tilde{v}}_{j}^{h}}$$

where $C_{{\hat{v}}_{i}^{h},{\tilde{v}}_{j}^{h}}$ is the original capacity of the edge, and $F_{{\hat{v}}_{i}^{h},{\tilde{v}}_{j}^{h}}$ represents the flow added to the edge during the current time slot.

**Figure 4:**
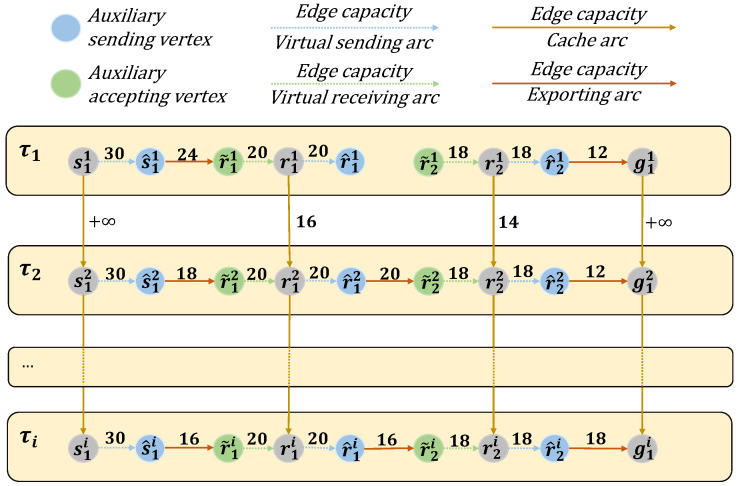
The process of dynamic updating in a dynamic satellite network, where $\tau_{i}$ represents the next time slot, and the network within the box corresponds to the network that will be updated in the next time slot. The residual network is indicated by the orange dashed box.

Figure 4 illustrates this update mechanism. The figure shows the residual network at a given time slot, the newly added snapshot graph for the next time slot, and how the two are merged to form the updated e-TEG. This process ensures that the residual network accurately reflects the current topology, resource capacities, and flow conditions of the LEO satellite network.

### 3.6. Predictive Maximum Flow in e-TEG

To efficiently compute the maximum flow in a dynamic LEO satellite network, we leverage a predictive flow $\hat{f}$ as an initial estimate to reduce the number of iterations needed to reach convergence, thereby accelerating the solving process.

The predictive maximum flow problem is defined on the e-TEG $G=(V^{*},A^{*})$, which represents the network’s topology across a time horizon $T=[t_{0},t_{k})$. The objective is to calculate the maximum flow *f* from the source satellite $s_{1}^{1}$ to the ground station $g_{1}^{h}$ while satisfying the following constraints:

(15) $$\begin{array}{cc} 0\leq f(\mu ,\nu )\leq c(\mu ,\nu ), & \;\forall \left(\mu ,\nu \right)\in A^{*} \end{array}$$

(16) $$\begin{array}{cc} F=\sum_{\mu \in V^{*}\setminus \left\{s_{1}^{1}\right\}}f\left(s_{1}^{1},\mu \right) & \end{array}$$

(17) $$\begin{array}{cc} \sum_{\left(\nu ,\mu \right)\in A^{*}}f\left(\nu ,\mu \right)=\sum_{\left(\mu ,\omega \right)\in A^{*}}f\left(\mu ,\omega \right), & \;\forall \mu \in V^{*}\setminus \left\{s_{1}^{1},g_{1}^{h}\right\} \end{array}$$

(18) $$\begin{array}{cc} \sum_{\left(\nu ,\mu \right)\in A^{*}}\hat{f}\left(\nu ,\mu \right)=\sum_{\left(\mu ,\omega \right)\in A^{*}}\hat{f}\left(\mu ,\omega \right), & \;\forall \mu \in V^{*}\setminus \left\{s_{1}^{1},g_{1}^{h}\right\} \end{array}$$

Here, f(μ,ν) denotes the data flow transmitted or stored on edge $\left(\mu ,\nu \right)\in A^{*}$, while c(μ,ν) represents the capacity of the edge. Equation (15) ensures that the flow on each edge does not exceed its capacity. Equation (16) defines the total flow *F* from the source $s_{1}^{1}$ to the ground station $g_{1}^{h}$, which is the objective to maximize. Equation (17) enforces flow conservation, ensuring that the total inflow to any intermediate node equals its total outflow.

The predictive flow $\hat{f}$ provides an initial estimate that satisfies flow conservation, as described in Equation (18). By initializing the algorithm with $\hat{f}$, the solver can begin with a near-feasible solution, reducing the number of iterations needed to compute the optimal maximum flow.

This predictive framework offers a significant computational advantage in dynamic networks, where frequent updates are required. The ability to incorporate predictive flows into the e-TEG model ensures that the computation remains efficient, even as the network evolves over time.

By employing this update mechanism, the e-TEG remains adaptable to the dynamic environment of LEO networks. The approach minimizes computational overhead by building on previously computed residual networks rather than reconstructing the graph from scratch for each time slot. This efficiency is critical for real-time applications requiring rapid response to changing network conditions.

### 3.7. Satellite Network Failures

The dynamic nature of LEO satellite networks is inherently susceptible to various disruptions, including satellite failures, link outages, and interference from cosmic events. These disruptions can significantly alter the network topology, necessitating a model that accurately reflects these changes. Key challenges include the intermittent disconnection of inter-satellite links (ISLs) due to satellite movement, orbital decay, or technical failures, as well as the limited lifespan of satellites. Additionally, other issues like beam pointing errors, insufficient channel capacity, and environmental factors like cosmic radiation further exacerbate the situation [37].

To address these potential disruptions, we propose a failure model that simulates the disconnection of ISLs due to unexpected events, providing a more robust framework for dynamic network changes. The failure model incorporates a time-slotted system where each time slot represents a discrete interval during which the network’s state can change due to these disruptions. For any ISL failure, we define a mapping function $\varphi_{k,h}:L_{h}\to L_{h+1}$ that adjusts the set of available ISLs from time slot $\tau_{h}$ to $\tau_{h+1}$, reflecting the link disconnection. If an ISL $\left(v_{i},v_{j}\right)$ fails in time slot $\tau_{h}$, it is removed from the set of available ISLs, $L_{h}$, **that is**, $L_{h+1}=L_{h}\setminus \left\{\left(v_{i},v_{j}\right)\right\}$. The new graph $G_{h+1}=\left(V,L_{h+1}\right)$ represents the updated network, capturing the new set of available links.

In response to unavailable links, the e-TEG graph adaptively adjusts the network topology and recalculates the maximum flow, ensuring that the flow optimization process remains accurate under changing conditions. Building upon the construction and updating techniques of the e-TEG model, Section 4 introduces the predictive acceleration algorithm, which optimizes flow calculations in the face of dynamic network changes.

## 4. Predictive Acceleration Algorithm for e-TEG Satellite Networks

This section presents methods for efficiently solving the maximum flow problem in e-TEG networks by using predictive flows and residual network updates. Predictive flows provide an informed starting point for computations, reducing the time needed to reach the optimal solution. Residual network updates enable efficient incorporation of dynamic changes in the network topology and resource constraints. To further enhance the adaptability and efficiency of our approach, we integrate an SDSN framework. By decoupling the data and control planes, SDSNs enable centralized decision-making, which improves routing flexibility and resource management.

### 4.1. Acquisition of Predictive Flow

Predictive flow is an informed estimate of the maximum flow for the current network state, derived from prior network configurations or heuristic approximations. The predictive flow enables a warm start for the algorithm, significantly reducing computational overhead.

To compute predictive flow in an e-TEG network G=(V,A) for a given time slot $\tau_{h}$, the algorithm identifies a feasible path from the source $s_{1}^{h}$ to the ground station $g_{1}^{h}$ while satisfying constraints in Equations (15) and (18) in Section 3. The predictive flow value $\hat{f}$ is then initialized as the minimum capacity along the path.

**Figure 5:**
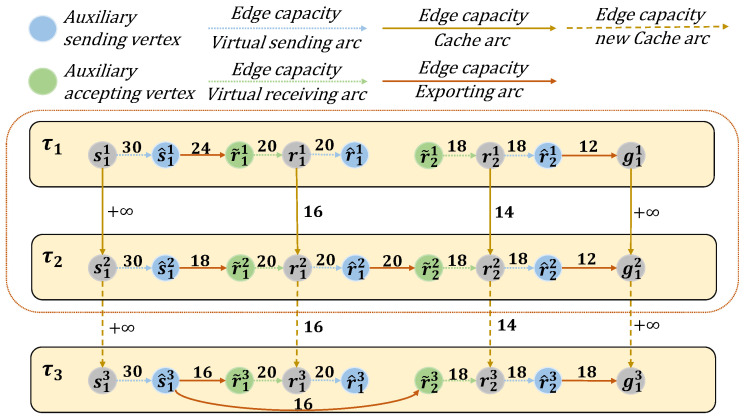
A time-varying e-TEG satellite network model with one observation satellite, two relay satellites, and one ground station spanning time slots $\tau_{1}$, $\tau_{2}$, and an additional slot $\tau_{3}$.

Figure 5 presents a demo example to illustrate an e-TEG satellite network operating within (2+1) consecutive time slots. This network comprises a source satellite, two relay satellites, and a ground station, with the time dimension divided into original time slots $\tau_{1}$ and $\tau_{2}$, as well as an additional time slot $\tau_{3}$. Since $s_{1}$ can transmit at any time, $C_{s_{1}^{h},s_{1}^{h+1}}$ is set to infinity, ensuring the existence of a path from $s_{1}^{1}$ to $s_{1}^{h}$. Therefore, the task of computing a predictive flow from $s_{1}^{1}$ to $s_{1}^{h}$ reduces to finding the predictive flow from $s_{1}^{h}$ to $g_{1}^{h}$.

To obtain the predictive flow for the e-TEG network for a given time slot, the algorithm begins by identifying a feasible path from $s_{1}^{h}$ to $g_{1}^{h}$, which is termed the predictive path. The algorithm then computes the flow capacity between each pair of nodes along this path, deriving the maximum flow value sustainable on it. This value serves as the predictive flow for the specified time slot, thereby defining the predictive flow for the e-TEG network. Details of the algorithm are presented in Algorithm 1.
**Algorithm 1** Acquisition of predictive flow in the e-TEG network.**Require:** Time-expanded graph $F_{h}=\left(V_{h},A_{h}\right)$, source $s_{1}^{h}$, and ground station $g_{1}^{h}$1:Initialize predictive flow vertex set $\tilde{V}\leftarrow V_{h}$, edge set $\tilde{A}\leftarrow \emptyset$, flow $\hat{f}\leftarrow \left(\tilde{V},\tilde{A}\right)$, and flow value δ←∞ {Initialize: preserve original nodes, reset edges, set bottleneck capacity to *∞*}2:Identify a path P from $s_{1}^{h}$ to $g_{1}^{h}$ {BFS to find the first feasible path satisfying Equations (15)–(18)}3:**for** each (μ,ν)∈P **do**4:   Add edge (μ,ν) to $\tilde{A}$ {Add edge to predictive flow network}5:   Update $\delta \leftarrow \min(\delta ,C_{h}\left(\mu ,\nu \right))$ {Track minimum capacity as bottleneck}6:**end for**7:**for** each (μ,ν)∈P **do**8:   Set edge flow capacity $\tilde{c}\left(\mu ,\nu \right)\leftarrow \delta$ {Set edge capacity to bottleneck value δ}9:**end for**10:**return** Predictive flow $\hat{f}$ {Return flow network with nodes, edges, and allocated capacities}

As shown in Algorithm 1, the algorithm begins with initialization (Step 1), where the predictive flow $\hat{f}$ is initialized with the graph’s vertex set $V_{h}$, an empty edge set $\tilde{A}$, and a flow value δ set to infinity. In Step 2, the algorithm commences a traversal using the breadth-first search (BFS) method starting from $s_{1}^{h}$, exploring all possible paths leading to $g_{1}^{h}$. For each candidate path identified during this traversal, it checks whether the path satisfies the constraints specified in Equations (15)–(18) in Section 3. Once the first feasible path is found during the search, it is selected as P. In the network shown in Figure 5, a feasible path P, namely $s_{1}^{3}\to {\hat{s}}_{1}^{3}\to {\tilde{r}}_{2}^{3}\to r_{2}^{3}\to {\hat{r}}_{2}^{3}\to g_{1}^{3}$, is identified from the source $s_{1}^{3}$ to the ground station $g_{1}^{3}$. During Steps 3–6, each edge (μ,ν), such as $\left(s_{1}^{3},{\hat{s}}_{1}^{3}\right)$, $\left({\hat{s}}_{1}^{3},{\tilde{r}}_{2}^{3}\right)$, $\left({\tilde{r}}_{2}^{3},r_{2}^{3}\right)$, $\left(r_{2}^{3},{\hat{r}}_{2}^{3}\right)$, $\left({\hat{r}}_{2}^{3},g_{1}^{3}\right)$ in the path P is added to $\tilde{A}$, and δ is updated to reflect the minimum capacity $C_{h}\left(\mu ,\nu \right)$, which in this case is 16, along the path. In Step 8, the capacity of each edge in P is set to δ. Finally, in Step 10, the predictive flow $\hat{f}$ is returned, comprising the updated vertex set, edge set, and capacities.

*Feasibility analysis of predictive flow:* The correctness and feasibility of the proposed algorithm are proven by demonstrating that the predicted flow $\hat{f}$ satisfies the capacity constraints in Equation (15) and the flow conservation constraints in Equation (18).

In Steps 3–6, Algorithm 1 calculates the minimum allowable flow δ along the path P, defined as follows:

(19) $$\delta =\min\left(C_{h}\left(\mu ,\nu \right)\right),\forall \left(\mu ,\nu \right)\in P$$

where $C_{h}\left(\mu ,\nu \right)$ denotes the capacity of edge (μ,ν). Since the flow on each edge $F_{h}\left(\mu ,\nu \right)$ is limited by its capacity, that is,

(20) $$F_{h}\left(\mu ,\nu \right)\leq C_{h}\left(\mu ,\nu \right),\forall \left(\mu ,\nu \right)\in P$$

it follows that the minimum flow δ is also bounded by the edge capacities:

(21) $$\delta \leq C_{h}\left(\mu ,\nu \right),\forall \left(\mu ,\nu \right)\in P$$

In Step 8 in Algorithm 1, the algorithm assigns this minimum flow δ to each edge in the path P. As a result, in the final predictive flow $\hat{f}$, the flow on each edge satisfies the capacity constraint:

(22) $$F_{h}\left(\mu ,\nu \right)\leq C_{h}\left(\mu ,\nu \right),\forall \left(\mu ,\nu \right)\in P$$

Next, consider an arbitrary intermediate node *v* on the path P (excluding the source node $s_{1}^{h}$ and the ground station $g_{1}^{h}$). Since the algorithm assigns a uniform flow value δ to all edges in P, the inflow and outflow for any intermediate node *v* are equal:

(23) $$\sum_{\nu }f_{out}\left(v,\nu \right)=\sum_{\mu }f_{in}\left(\mu ,v\right)=\delta ,\forall v\in V\setminus \left\{s_{1}^{h},g_{1}^{h}\right\}$$

Thus, the predicted flow $\hat{f}$ satisfies both the capacity and flow conservation constraints, ensuring the feasibility of the algorithm.

*Complexity analysis:* The time complexity of this algorithm depends on two primary components: identifying the feasible path P and updating flow rates. The complexity of finding the path P depends on the graph traversal method. The use of BFS to identify a path has a time complexity of O(V+E), where *V* and *E* denote the number of nodes and edges, respectively. Once the path P is identified, the algorithm iterates through the edges in P to calculate the minimum flow δ and assign it to the edges. This step involves O(|P|) iterations, where |P| is the number of edges on the path. In the worst case, |P| can equal O(V) if the path passes through all nodes of the graph. Combining these components, the overall time complexity of the algorithm is O(V+E+|P|). In the worst-case scenario, where |P|=O(V), the complexity simplifies to O(V+E). This indicates that the algorithm is efficient and scales linearly with the size of the graph, making it well-suited for dynamic e-TEG networks.

### 4.2. Residual Network Update

In this subsection, we describe the process of updating the e-TEG network to account for the dynamic evolution of its topology as shown in Algorithm 2.
**Algorithm 2** Residual network update based on the e-TEG network.**Require:** Newly added network $G^{\prime }\left(V^{\prime },A^{\prime }\right)$, residual network G(V,A)1:$V^{*}\leftarrow V\cup V^{\prime }$ {Merge node sets from old and new networks}2:**for** each $\left(\mu ,\nu \right)\in A^{\prime }$ **do**3:   $A^{*}\leftarrow A\cup \left\{(\mu ,\nu )\right\}$ {Integrate new edges into residual network}4:   **if** $c^{\prime }\left(\mu ,\nu \right)>0$ **then**5:     $c^{*}\left(\mu ,\nu \right)=c^{\prime }\left(\mu ,\nu \right)$ {Update capacity for active edges}6:   **end if**7:**end for**8:For the observation satellite $s_{1}$, $c^{\prime }\left(s_{1},s_{1}^{\prime }\right)\leftarrow +\infty$ {Set infinite capacity for observation links}9:For the ground station $g_{1}$, $c^{\prime }\left(g_{1},g_{1}^{\prime }\right)\leftarrow +\infty$ {Ensure unbounded downlink capacity}10:**for** μ∈ all LEO satellites **do**11:   $c^{\prime }\left(\mu ,\mu^{\prime }\right)\leftarrow E_{\mu }$ {Assign energy-constrained capacity using $E_{\mu }$}12:**end for**13:**return** {Updated residual network $G^{*}\left(V^{*},A^{*}\right)$}

In Algorithm 2, we construct a time-unfolding graph G(V,A) to represent the e-TEG network from the initial time interval $\tau_{1}$ to the *i*-th time interval $\tau_{i}$. When the network expands into a new time interval, denoted as $\tau_{i+1}$, we construct a novel snapshot graph and transform it into an e-TEG network, represented as $G^{\prime }\left(V^{\prime },A^{\prime }\right)$. This snapshot graph captures the current state of the network at $\tau_{i+1}$, which includes newly introduced nodes, edges, and transmission paths. We then integrate this new snapshot graph into the existing time-unfolding graph to form an updated time-unfolding graph $G^{*}\left(V^{*},A^{*}\right)$, where in Step 1, $V^{*}=V\cup V^{\prime }$; in Step 3, $A^{*}=A\cup A^{\prime }$; and in Step 5, the capacity of each edge is updated. This process ensures that the evolving network topology is accurately captured and represented in the time-unfolding graph.

*Complexity analysis:* The proposed update process leverages the continuity of the time-unfolding graph, eliminating the need to reinitialize the network from scratch. Instead, the algorithm incrementally integrates the newly added components into the residual network from the previous interval. This approach significantly reduces computational overhead compared to traditional methods.

The time complexity of the update process is determined by the size of the newly added network components. Specifically, adding new edges requires $O(E^{\prime })$, where $E^{\prime }$ is the number of edges in the newly added network, adding new vertices requires $O(N^{\prime })$, where $N^{\prime }$ is the number of vertices in the newly added network, and updating capacities requires $O(E^{\prime })$, as edge capacities are modified based on the new snapshot. Thus, the overall time complexity for updating the network is $O(E^{\prime }+N^{\prime })$. This incremental update approach is considerably more efficient than traditional methods, which require reinitializing the entire network for every update. As a result, the algorithm is well-suited for dynamic e-TEG networks, where frequent topology changes must be handled efficiently.

### 4.3. Predictive Accelerated e-TEG Network Maximum Flow Algorithm

Here, we present an optimized maximum flow algorithm for dynamic e-TEG networks, leveraging predictive flow and residual network updates to enhance computational efficiency. The algorithm builds on the predictive flow obtained from the predictive flow acquisition algorithm (Algorithm 1) and the residual network structure maintained using the residual network update algorithm (Algorithm 2). By combining these components, the algorithm efficiently computes the maximum flow for a given time slot, adapting to changes in the network topology. The predictive flow provides a “warm start”, reducing the number of iterations required, while the residual network ensures that updates reflect the current network state.

The predictive accelerated maximum flow algorithm (Algorithm 3) integrates predictive flow and residual network updates to efficiently compute the maximum flow. The algorithm begins with initialization (Step 1), where the residual graph $G^{*}$ is set to the current network *G*, and the total flow $f^{*}$ is initialized by combining the predictive flow $\hat{f}$ and the maximum flow from the previous time slot, $f_{\mathrm{prev}}^{*}$. This initialization incorporates historical and predictive information to provide a strong starting point. Next, in Steps 2–4, the algorithm adjusts the capacities in *G* by subtracting the capacity already occupied by $\hat{f}$. This step ensures that the residual graph reflects the remaining capacity available for flow augmentation, effectively using the residual network update process from the previous algorithm. The algorithm then performs the Ford–Fulkerson computation on the adjusted residual graph *G* (Step 5). This step computes the additional maximum flow $f_{\mathrm{res}}$ that can be achieved given the residual capacities. By starting with a graph already adjusted for $\hat{f}$, the number of iterations required to converge is significantly reduced. In Steps 6–8, the algorithm updates the residual capacities in $G^{*}$ by subtracting the flows from both $f_{\mathrm{res}}$ and $\hat{f}$. This ensures that the final residual network $G^{*}$ is consistent with the total flow. Finally, the algorithm combines $f_{\mathrm{res}}$ and $\hat{f}$ to compute the total maximum flow $f^{*}$ (Step 9). The updated $f^{*}$ satisfies flow conservation and capacity constraints and is returned along with the residual graph $G^{*}$.
**Algorithm 3** Predictive accelerated maximum flow algorithm for e-TEG networks.**Require:** Updated network G(V,A), maximum flow $f_{\mathrm{prev}}^{*}\left(V^{*},A^{*}\right)$ from the previous time slot, predictive flow $\hat{f}\left(V_{\hat{f}},A_{\hat{f}}\right)$, source *s*, and sink *g***Ensure:** Maximum flow $f^{*}$ and updated residual network $G^{*}$1:Set residual graph $G^{*}\leftarrow G$, residual flow $f_{\mathrm{res}}\leftarrow \emptyset$, and total flow $f^{*}\leftarrow f_{\mathrm{prev}}^{*}+\hat{f}$ {Initialize the residual graph and total flow by combining historical and predictive flows}2:**for all** $\left(\mu ,\nu \right)\in A_{\hat{f}}$**do**3: Update residual capacity: $c_{G}\left(\mu ,\nu \right)\leftarrow c_{G}\left(\mu ,\nu \right)-c_{\hat{f}}\left(\mu ,\nu \right)$ {Adjust residual capacities to reflect the flow already occupied by the predictive flow}4:**end for**5:Run Ford–Fulkerson algorithm on *G* to compute residual flow $f_{\mathrm{res}}$ from *s* to *g* {Compute additional flow using the Ford–Fulkerson algorithm on the adjusted residual graph}6:**for all **(μ,ν)∈A **do**7: Update residual capacities: $c_{G^{*}}\left(\mu ,\nu \right)\leftarrow c_{G}\left(\mu ,\nu \right)-c_{f_{\mathrm{res}}}\left(\mu ,\nu \right)-c_{\hat{f}}\left(\mu ,\nu \right)$ {Update residual capacities to reflect both residual and predictive flows}8:**end for**9:Set total flow: $f^{*}\leftarrow f_{\mathrm{res}}+\hat{f}$ {Combine residual and predictive flows to obtain the total maximum flow}10:**return** $f^{*}$, $G^{*}$

*Complexity analysis:* The time complexity of the predictive accelerated maximum flow algorithm depends on the relationship between the predictive flow $\hat{f}$ and the optimal flow $f_{c}^{*}$ on the graph *G*. Using the predictive flow $\hat{f}$ as the initial seed significantly reduces the number of iterations required to compute the maximum flow compared to starting from zero.

The Ford–Fulkerson algorithm operates iteratively, augmenting the flow value until it reaches the maximum flow. Each iteration of the algorithm finds an augmenting path and sends flow along it, requiring O(E) time, where *E* is the number of edges in the graph. The number of iterations is proportional to the difference in flow values between the predictive flow and the optimal flow, specifically at most $val\left(f_{c}^{*}\right)-val\left(\hat{f}\right)$ iterations.

In Steps 2–4, the algorithm adjusts the residual capacities of edges based on the predictive flow $\hat{f}$. This operation requires $O(E_{\hat{f}})$ time, where $E_{\hat{f}}$ is the number of edges in the predictive flow. In Step 5, the Ford–Fulkerson algorithm computes the residual flow $f_{\mathrm{res}}$ by iterating over augmenting paths. For each augmenting path, the runtime is O(E), and the number of iterations is bounded by $val\left(f_{c}^{*}\right)-val\left(\hat{f}\right)$. Hence, the runtime of this step is as follows:

(24) $$O(E\cdot \left(val\left(f_{c}^{*}\right)-val\left(\hat{f}\right)\right))$$

In Steps 6–8, the algorithm updates the final residual capacities by subtracting the flows of $\hat{f}$ and $f_{\mathrm{res}}$. This step requires O(E) time.

Combining these components, the total runtime is dominated by the Ford–Fulkerson computation and can be expressed as follows:

(25) $$O(E\cdot \left(val\left(f_{c}^{*}\right)-val\left(\hat{f}\right)\right)+E)$$

which simplifies to $O(E\cdot \left(val\left(f_{c}^{*}\right)-val\left(\hat{f}\right)\right))$ in practical scenarios where $val\left(f_{c}^{*}\right)-val\left(\hat{f}\right)\gg 1$.

Let $\eta =\left|\right|\hat{f}-f_{c}^{*}{\|}_{1}$ represent the prediction error, which quantifies the difference between the predictive flow $\hat{f}$ and the optimal flow $f_{c}^{*}$. This error provides an upper bound on the number of iterations required by the algorithm, as $val\left(f_{c}^{*}\right)-val\left(\hat{f}\right)\leq \eta$. Therefore, the runtime can be further bounded by the following:

(26) O(E·η)

where η reflects both the quality of the predictive flow and the efficiency of the algorithm. The quality of the predictive flow $\hat{f}$ is measured as follows:

(27) $$\eta \left(\hat{f}\right)=\min_{f^{*}\in F^{*}}\|\hat{f}-f^{*}{\|}_{1}$$

where $F^{*}$ represents the set of all possible maximum flows. In cases where there are multiple maximum flow solutions, $\eta (\hat{f})$ is calculated with respect to the closest solution in $F^{*}$. The smaller the prediction error $\eta (\hat{f})$, the fewer iterations are required to reach the optimal flow, and thus, the faster the algorithm converges.

The runtime of the algorithm depends linearly on the size of the graph (that is, *E*) and the prediction error η. By leveraging the predictive flow $\hat{f}$, the algorithm significantly reduces the number of iterations required to compute the maximum flow. This makes the algorithm particularly efficient for dynamic e-TEG networks, where predictive flows can be updated frequently to match the evolving topology.

### 4.4. SDSN

Incorporating predictive acceleration into e-TEG networks improves computational efficiency, but real-time network adaptation remains a challenge. To address this, we leverage the SDSN framework, which enhances network programmability and flexibility.

SDSN [38] is a transformative approach in satellite communication networks, leveraging SDN principles to separate data and control planes. This minimizes hardware demands and focuses resources on data transmission, while control plane satellites enable real-time network status updates for efficient policy deployment and resource scheduling.

We implement a satellite routing algorithm within the SDSN framework to enable dynamic flow control and intelligent network adaptation. In the SDSN framework, we have the management plane based at the ground center, which computes the global routing paths and predicts flow based on real-time information from the LEO satellites and GEO satellites. GEO satellites, as network controllers, provide global oversight, compiling a comprehensive view of the LEO network and enabling real-time updates to routing paths. The data plane comprises LEO satellites and ISLs, responsible for packet forwarding based on programmed forwarding tables provided by the management plane. By implementing the proposed algorithms within the SDSN framework, LEO satellites follow the optimized paths computed by the predictive acceleration algorithm, reducing computational overhead and ensuring that flow optimization is dynamically adapted to real-time network changes.

## 5. Evaluations

To evaluate the performance of the predictive acceleration algorithm for maximum flow in e-TEG networks, we utilized the system tool kit (STK) [39] to model SpaceX’s Starlink [1] satellite constellation. This constellation comprises satellites orbiting at an altitude of 550 km with a 53-degree inclination, distributed across five orbital planes.

Given the computational complexity of simulating the full Starlink constellation, we adopt a scalable evaluation framework using 10, 15, 20, and 25 satellites. While these configurations represent a fraction of the full network, they preserve key topological properties of large-scale LEO constellations, including dynamic connectivity and constrained onboard resources. Prior work in temporal graph analysis and network optimization demonstrates that reduced-scale networks effectively capture algorithmic behavior while enabling controlled, repeatable experimentation [11,40].

To establish a representative baseline for constellation construction, we first identified a canonical Starlink satellite (550 km altitude, 53° inclination) from operational TLE data as the orbital seed. Using this seed as the template, Walker Delta [41] constellations were programmatically generated in STK by replicating its characteristics thereby preserving Starlink-like topological properties. The architecture was implemented with precise orbital parameters, including phase offsets optimized for collision avoidance and uniform coverage. Satellite trajectories were propagated using the SGP4 model [42].

To capture realistic network dynamics, we established a 1 h evaluation window starting at 04:00 UTC on 14 October 2024, divided into twelve 5 min intervals corresponding to characteristic LEO network state changes. Link configurations were rigorously modeled with fixed 1 Gbps data rates for both ISLs and ground-to-satellite links (GSLs), incorporating practical constraints. Onboard storage capacities were parameterized across three configurations (500 GB, 600 GB, 700 GB) to systematically evaluate resource-constrained adaptation capabilities.

We compared the proposed predictive acceleration algorithm against existing maximum flow methods for e-TEG networks, including the standard Dinic’s algorithm [8], MPTPT-based routing [43], and the e-TEG algorithm [11]. In Dinic’s algorithm, augmenting paths are found using BFS to construct a layered network, and the depth-first search is employed to send flow along these layers. MPTPT-based routing iteratively selects the most promising path based on current conditions, optimizing flow based on the immediate network state. The e-TEG algorithm extends the traditional time-expanded graph framework by incorporating energy constraints, effectively modeling satellite networks with time-varying topologies and dynamic link capacities.

To evaluate the computational efficiency of the proposed algorithm and other existing algorithms, we use running time as the main metric, defined as the time taken to compute the maximum flow. In addition to running time, we also introduce the acceleration percentage to quantify the reduction in computational effort relative to the e-TEG algorithm. This metric compares the performance of the evaluated algorithm with the e-TEG algorithm as the baseline. The acceleration percentage is defined as follows:

(28) $$AccelerationPercentage=\frac{t_{e-TEG}-t_{compared}}{t_{e-TEG}}$$

where $t_{e-TEG}$ represents the running time of the e-TEG algorithm, and $t_{compared}$ is the running time of the algorithm being evaluated.

**Figure 6:**
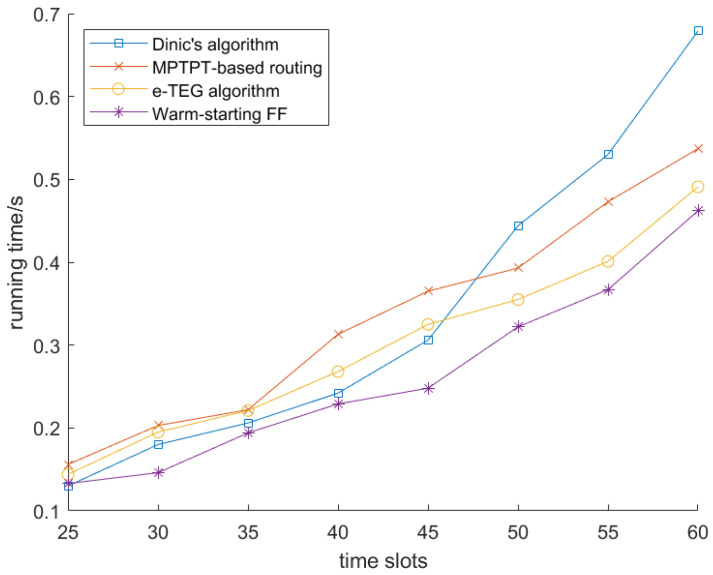
Running time comparison of warm-starting FF.

**Figure 7:**
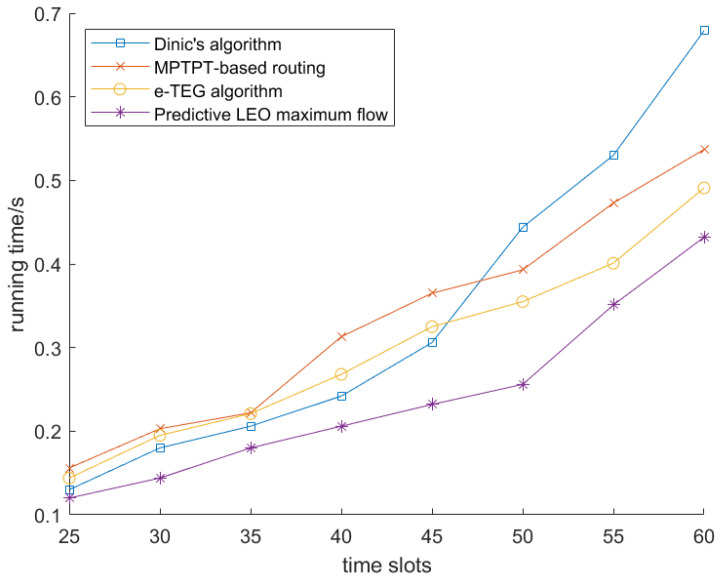
Running time comparison of predictive LEO maximum flow.

We first analyzed the running time of the warm-starting Ford–Fulkerson algorithm (labeled as “warm-starting FF” in the figures) and the predictive LEO maximum flow algorithm alongside three existing methods in Figure 6 and Figure 7, respectively. For this analysis, we used a satellite network consisting of 25 satellites, each with a limited storage capacity of 600 GB. The running times of the four algorithms were evaluated as the number of time slots varied from 25 to 60.

As shown in Figure 6, the running time of all four algorithms increases as the number of time slots rises. However, the proposed warm-starting Ford–Fulkerson algorithm consistently exhibits the smallest running time compared to the other algorithms. This is mainly because the warm-starting Ford–Fulkerson algorithm leverages previously computed flow information from earlier time slots, significantly reducing the time needed for flow calculations in subsequent iterations. In contrast, the e-TEG algorithm performs worse than Dinic’s for a smaller number of time slots because it requires additional overhead to update and maintain the time-expanded graph, leading to longer computation times. However, as the number of time slots increases, e-TEG benefits from its energy constraints and dynamic topology adaptation, allowing it to outperform Dinic’s algorithm in more complex scenarios. We further calculated the acceleration percentage of the warm-starting Ford–Fulkerson algorithm, shown in Table 2, where it reached a peak of 32.24%.

Figure 7 presents a comparative analysis of the acceleration performance between the existing algorithms and the predictive LEO maximum flow algorithm. Notably, the predictive LEO maximum flow algorithm demonstrates a significant reduction in overall running time. This is primarily because predictive LEO maximum flow not only leverages predictive flow acquisition (that is, estimating flow based on past network states) but also incorporates dynamic adaptability to changing network conditions, allowing it to make more efficient routing decisions and minimize the need for recalculations. However, it is slightly less efficient than warm-starting FF in some cases because warm-starting FF directly utilizes historical flow data from earlier time slots, leading to faster convergence and fewer recalculations when network topology changes incrementally. In contrast, predictive LEO maximum flow, while highly efficient, sometimes needs to adjust more dynamically in response to more substantial changes in the network. Table 2 further provides the acceleration percentages achieved by the predictive LEO maximum flow algorithm across varying numbers of time slots. As shown in Table 2, the predictive LEO maximum flow algorithm consistently improves in performance, reaching a peak acceleration of 23.36%.

**Figure 8:**
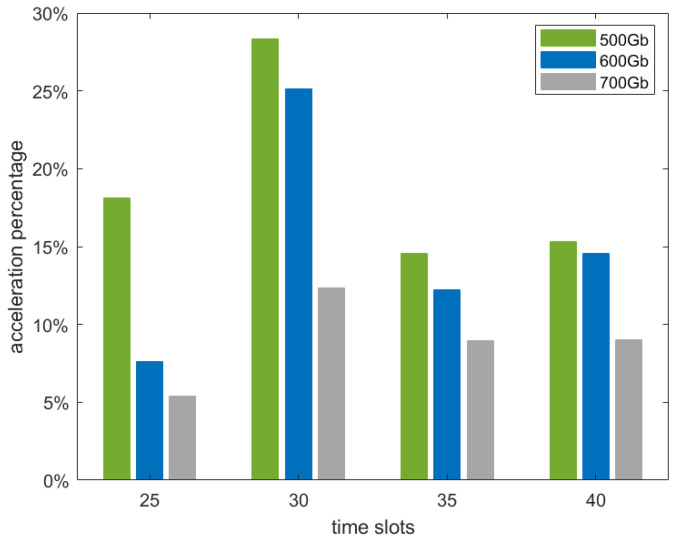
Acceleration performance of predictive LEO maximum flow with varying satellite storage capacities.

**Figure 9:**
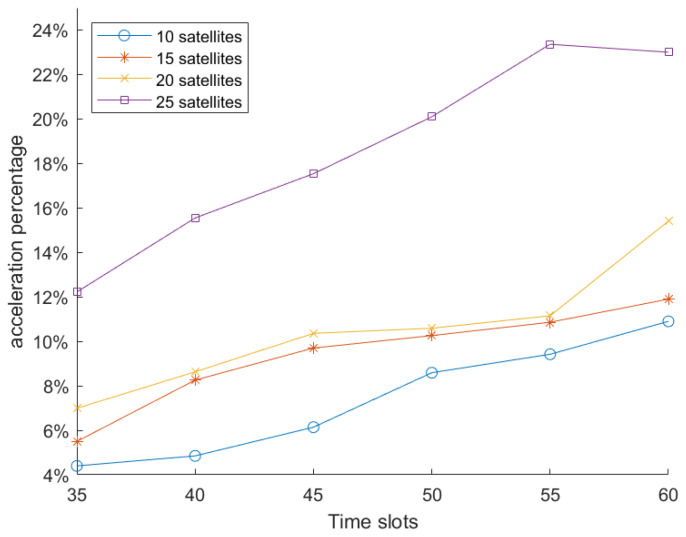
Acceleration performance of predictive LEO maximum flow with a varying network scale.

We also considered the impact of satellite storage capacity on algorithm performance, as well as the effect of network scalability in terms of the number of satellites, as shown in Figure 8 and Figure 9, respectively.

Figure 8 presents the acceleration percentage of predictive LEO maximum flow for different storage configurations (500 GB, 600 GB, and 700 GB) as the number of time slots varies from 25 to 40. As observed in Figure 8, when available storage capacity decreases, the predictive LEO maximum flow algorithm achieves a higher acceleration percentage. This is because lower storage capacity imposes stricter resource constraints, compelling the algorithm to more effectively utilize its predictive capabilities. With less storage available, the algorithm must make better decisions about data flow management, ensuring more efficient use of computational resources while maintaining high flow throughput. This trend suggests that stricter resource constraints incentivize the algorithm to focus on the most efficient flow allocation strategies within the bounded computational budgets, leading to improved performance in terms of both computational efficiency and flow optimization.

Figure 9 shows the changes in the acceleration percentage of predictive LEO maximum flow as the network scale increases from 10 to 25 satellites. In this evaluation, we fixed the storage limitation on each satellite to 600 GB and varied the number of time slots from 35 to 60. When the network scale increases, the acceleration percentage increases as well, indicating that the predictive LEO maximum flow algorithm becomes more efficient in larger and more complex LEO networks. This is because larger networks provide more opportunities for predictive flow allocation and allow the algorithm to optimize paths across multiple time slots, thereby reducing the overall computational effort. As the number of satellites increases, the algorithm can better adapt to dynamic changes in the network, leveraging its predictive capabilities to efficiently manage larger volumes of data and more complex topologies, leading to an overall improvement in performance.

## 6. Conclusions and Future Work

In this paper, we introduced a predictive acceleration algorithm for solving the maximum flow problem in LEO satellite networks. By leveraging a predictive warm-start approach, our algorithm significantly enhances the efficiency of traditional flow algorithms, enabling faster recalculations of maximum flow in dynamic, resource-constrained environments.

Our extensive evaluation demonstrated that the predictive flow acquisition mechanism, along with the warm-starting Ford–Fulkerson approach, delivers substantial acceleration in maximum flow computation, especially under varying network scales and storage constraints. The proposed predictive LEO maximum flow algorithm consistently outperforms existing methods such as Dinic’s algorithm, MPTPT-based routing, and the e-TEG algorithm, particularly in terms of computational efficiency. The acceleration percentages achieved by the proposed algorithm, especially in scenarios with constrained satellite storage, highlight the algorithm’s ability to optimize flow allocation while respecting dynamic network conditions.

In the future, we will aim to further optimize the pathfinding process to enhance real-time responsiveness in large-scale satellite networks. Currently, we use BFS for predictive flow acquisition, which has a time complexity of O(V+E). While the proposed warm-starting FF and predictive LEO maximum flow algorithms already demonstrate superior time efficiency compared to existing methods, full BFS may introduce non-negligible overhead in scenarios involving larger constellations or more frequent topology changes. Therefore, we plan to further reduce BFS overhead and enhance computational speed. One direction for improvement could involve integrating advanced data structures, such as dynamic tree architectures, to streamline path optimization. We also plan to investigate depth-limited or priority-guided BFS strategies that can reduce search overhead while preserving accuracy. Furthermore, we plan to incorporate machine learning-based traffic prediction models, such as temporal graph neural networks and adaptive reinforcement learning strategies, to improve the accuracy and responsiveness of flow forecasting in dynamic satellite networks. Finally, we will develop distributed computing paradigms to ensure scalability for mega-constellation deployments.

## Figures and Tables

**Table 1:** Comparison of TEG/e-TEG models and representative maximum flow algorithms.

Method	Characteristics	Advantages	Limitations
TEG [11]	Models the dynamic topology of satellite networks using time-based snapshots.	Captures temporal variation in connectivity; supports time- aware routing.	Ignores energy, processing, or bandwidth constraints.
e-TEG [11]	Extends TEG by incorporating energy constraints using virtual arcs.	Addresses energy limitations; enables energy-aware flow optimization.	Increased model complexity; computationally intensive.
Ford–Fulkerson Algorithm [7]	Iteratively finds augmenting paths until no residual capacity exists.	Simple; guarantees max flow in integral capacity networks.	Slow for large or dynamic networks; recomputation required after topology changes.
Dinic’s algorithm [8]	Uses layered networks and blocking flows for efficient maximum flow computation.	Lower time complexity $\left(O\left(n^{2}m\right)\right)$, suitable for large-scale networks.	Assumes static topology; complex implementation.
Learning-augmented maximum flow algorithm [9]	Uses predicted flow values to accelerate computation, leveraging prior knowledge.	Reduces augmentation steps; adapts to recurring flow patterns.	Prediction quality affects stability.
0–1 Min-cost flow algorithm [17]	Solves min-cost flow under binary capacity and cost constraints.	Efficient for highly structured, restricted problems.	Not directly applicable to general or dynamic satellite topologies.
Approximate maximum flow algorithm [24,25]	Computes approximate maximum flows for large-scale networks.	Fast computation; scalable to large networks.	Approximation error; less accurate in constrained or real-time settings.
Near-linear maximum flow algorithm [26]	Uses data structures and optimization techniques to achieve near-linear complexity.	Theoretically optimal for very large networks.	Assumes static input; lacks support for real-time adaptability.

**Table 2:** Acceleration percentage of warm-starting FF and predictive LEO maximum flow compared to e-TEG.

Time Slots	Acceleration Percentage	
	**Warm-Starting FF**	**Predictive LEO Maximum Flow**
25	16.67%	7.64%
30	26.15%	25.13%
35	18.55%	12.22%
40	23.13%	14.55%
45	28.62%	17.54%
50	31.51%	20.10%
55	32.24%	23.36%
60	28.00%	23.00%

## Data Availability

The original contributions presented in this study are included in the article. Further inquiries can be directed to the corresponding author.
